# Two Meetings at St. Paul

**Published:** 1901-07

**Authors:** 


A Monthly Review of Medicine and Surgery.

EDITOR:

WILLIAM WARREN POTTER, M. D.

All communications, whether of a literary or business nature, books for review and exchanges should be addressed to the editor: 284 FRANKLIN STREET, BUFFALO, N. Y.

VOL. XL.—LVI. JULY, 1901. NO. 12.

### TWO MEETINGS AT ST. PAUL.

IN JUNE, 1882, at the meeting of the American Medical Association at St. Paul, a tragedy was enacted which shocked the medical profession of the country so grievously that it has not yet recovered from the results thereof. Briefly stated, the facts are these: The Medical Society of the State of New York had then lately modified the code of medical ethics, to make it conform to the statutory laws from which the society derived its authority. The enemies of this reform have unjustly, if not maliciously, charged that its object was to permit consultations with homeopaths and other unrecognised sects in medicine. Nothing was further from the truth. The sole and entire object of the modification of the code was to remove antagonism between the by-laws of the society and the statutes of the state. This enabled the society to obtain from the legislature what is concededly one of the best practice acts in operation in the United States.

The society elected thirty-five of its most representative men as delegates to the St. Paul meeting in 1882. Dr. Cornelius R. Agnew, one of the most amiable as well as learned physicians, was chairman of the delegation, and when he arose to state the facts in the case to the association, his mouth was ruthlessly shut by the action of that body, which declined to hear one word in extenuation or explanation. Even the commonest criminal is granted the privilege of defending himself at the bar of justice. The treatment of Dr. Agnew and his colleagues was cruel, to say the least, and we have never yet heard or read any successful attempt to defend it.

Nineteen years afterward, that is to say in June, 1901, the American Medical Association met again at St. Paul, but in a different atmosphere and with a different environment. The president, Dr. Charles A. L. Reed, of Cincinnati, is a man of such liberal training and of such broad views, that it was inevitable that something should be done under his administration to right the great wrong committed in 1882, and to correct the great evil which resulted therefrom. That several other states had violated the so-called code, which some of the old-time leaders of the association pretend to love so dearly, without having been disciplined for their action as had New York, is a well-known fact; but it remained for Dr. Reed to show up this inconsistency in his annual address, in a fashion that must have made some of the men referred to experience a qualm of shame for their inconsistency.

Dr. Reed's address is a masterful state paper, which should be read by every member of the association and carefully studied, especially by the younger members. It deals with every phase of the association's work from its inception, and records its successes and failures with a fidelity to truth that no previous president has had the courage to delineate. Many of its recommendations relating to scientific investigation and the advancement of professional interests are deserving of the most patient thought by scientific men.

In accomplishing the reorganisation of the association, however, Dr. Reed has done the American medical profession an act of kindness that will outlast the work of every one of his predecessors. In establishing the house of delegates to take charge of the legislative work, in practically abolishing the offensive features of the code, and in proclaiming a unity of sentiment in the profession, he has lifted the association up to a higher plane than it yet has ever occupied. To have accomplished so much in a single year of administrative life stamps him with a most honorable fame, and entitles him to the gratitude of every American physician.

---

THE following excerpts from President Reed's presidential address at Saint Paul, will repay careful reading by every friend of medical progress:

At the same time, however, it became apparent that the organisation did not possess the necessary inherent strength to accomplish its avowed object to regulate the practice of medicine. As time passed schismatic medicine grew apace, its colleges mutiplied, its practitioners appeared all over the country exemplifying that law that always makes the blood of the martyrs the seed of the church. Quackery of the most flagrant character was found everywhere, and society was unprotected from its ravages, while the inability of a voluntary unchartered organisation to enact and to execute plenary laws was reduced to a demonstration. The medical profession, as an organised body, discovered that its relation to the commonwealth was, as the result of its own proscriptive policy, scarcely more intimate or more influential than at the beginning of the thirty years' hopeless experiment.

The profession in each state, however, recognising its own local conditions, proceeded in its own way to attend to its own business. The very earliest attempt to secure state legislation revealed the fact that the so-called irregular practitioners, under the stimulus of ostracism and the fostering care of public sympathy thereby induced, had become so numerous and influential that in the majority of states nothing could be done without their coöperation. It was no longer a theory, but a condition with which the real reformers were confronted —and they met it. California, in 1876, through its regular medical society, took the initiative. After conferences with the representatives of the sectarian societies, and after securing their coöperation, a law was procured, creating a licensing board composed of representatives of both the regular and sectarian schools of practice. Illinois, confronted by precisely the same condition, took precisely the same course. Alabama, always progressive, but the happy possessor of other conditions, was able to place the regulation of medical practice for the time being under the control of its incomparable state medical association. Colorado created a mixed board. New York, confronted by conditions even more complicated than those in other states, took up the same task. The profession of that state, acting through its organised body, containing among its members many of the most honored and illustrious names in American medicine, found it doubly necessary to enter into treaty with the denominational physicians. It realised, however, that the rules of conduct to which it had always conformed contained among other provisions one which made it unlawful to " . . examine or sign diplomas or certificates of proficiency for, or otherwise be especially concerned with the graduation of persons whom they have good reason to believe intend to support and practice any exclusive and irregular system of medicine."

As the thing expressly interdicted by this rule was the very thing which it proposed to do, and which had been done in other states, and which it was very necessary to do in New York, the medical society of that state amended the rules of conduct so that it or its members might, at discretion, enter into professional relations with any or all persons whom the law of the state at that time recognised to be practitioners of medicine. When this action was brought to the attention of this national body it resulted, not as might have been expected, in the amendment or the abrogation of the rule which had grown obsolete in the march of events, but in its tacit reaffirmation and in the opprobrious excommunication, for the time being of the entire profession of the great Empire state. This action, viewed impartially after the lapse of nearly twenty years, becomes the more extraordinary when it is observed that similar action was never taken with regard to Massachusetts or Rhode Island or Mississippi, the societies of neither of which had ever adopted the prescribed rules of conduct; nor with regard to California or Illinois or Colorado, each of which had, by overt act, if not by open declaration, so far as this rule is concerned, taken an equally non-conformist position. It is not surprising that, with such an example before the state societies, the experiment in consistency has not been repeated. But the movement of effective regulative legislation, once inaugurated, happily spreads with great rapidity. Mixed boards of licensure are now to be found in the majority of the states of the Union, and in the majority of such boards are to be found members of the American Medical Association engaged in issuing licenses to practitioners of exclusive dogmas, and sitting in consultation with sectarian physicians, not over a dose of medicine, but over the more vastly vital question of the qualifications of those who are to care for the sick of our Republic.

Today there are forty-eight state or territorial licensing boards, the most of them being composed of representatives of both the regular and the sectarian schools of practice. The laws of the different states are of varying efficiency, the one procured by the Medical Society of the State of New York, at the price of yet maintained excommunication from this body, standing today as the model of excellence for the entire country.

We thus see that the proscriptive rule which, during the more than twenty-five years of its dominance, propagated the very evils it was intended to correct, is rapidly expiring by limitation in the face of new conditions that have been induced, in spite of it, by beneficent and catholic legislation. In the State of New York alone the annual registration of sectarian physicians has diminished nearly 90 per cent. under the operation of its present laws.

The committee on reorganisation, under the restrictions of the resolution creating it, has, very properly, left undisturbed the existing rules of conduct. These, if construed to have a fundamental importance, and if vigorously enforced as they now stand, would disintegrate the association in a single day. This reason, and others already given, confirm me in the conviction that such rules should be either amended or abrogated, or, if reaffirmed, it should be by general resolution endorsing their underlying principles but disclaiming the present applicability of their details.

---

A CENSUS of the consumptives in this state is to be begun very soon by Dr. Daniel Lewis, state commissioner of health. It will be the first census of the kind ever undertaken in this state. The census is for the purpose of ascertaining the number of consumptives in the state as far as possible, and revealing other facts relating to the disease. It is expected that this enumeration will throw light on the question of what the state should do for the care of those within its borders who are afflicted with consumption, and who cannot afford to pay for treatment at the private sanitoriums.

About eighteen or twenty months after this census is completed another will be taken and the results of the two will form, it is thought, a good basis for comparison. The results of these statistical tables will reveal to the commissioner of health and to the public whether consumption is on the increase or not as well as other valuable facts.

---

THE JOURNAL has received the following communication, which explains itself:

CINCINNATI, June, 1901.

I am endeavoring to establish the facts which I believe from my own personal observation to be true (see *Lancet Clinic*, June 8, 1901,) of an inherited tendency or predisposition to appendicitis. If the readers of your journal will kindly look into the history (family) of their cases and report results to me, I will be under very many obligations.

W. H. DEWITT, M. D.,
61 Auburndale Place.

---

THE New York *Tribune* of May 28, 1901, says: Dr. Joseph Price, in a paper which he read before the Philadelphia County Medical Society, the other day said: "I attribute the enormous increase of appendicitis among women to golf, cricket, the bicycle and other outdoor sports, which at times subject them to prolonged physical exertion and inclement weather."

---

DEAR old Rip Van Winkle, says the *Post-Graduate*, is put completely in the shade by a recent article in the *Jour. N. Y. State Med. Assn.*, entitled, The Reason for the Existence of the New York State Medical Association. The author explains in seven columns why the association seceded from the state medical society. It was all because the majority of the state medical society for three years in succession, deliberately decided that its members might consult with any legally qualified practitioner. Without this decision there would have been no law creating a state board of medical examiners, as the

*Post-Graduate* has so often shown, but our friend, the author of this antediluvian history, seems to think it a very reasonable thing that a society should be excluded from the American medical association because it conforms to the law of the state, does not pretend to be wiser than the state authorities, and permits its members to consult with any one that the authorities have decided to be a legal practitioner.

